# Operational Lessons From Implementing Public Health Data–Based Recruitment and Home STI Screening to Enroll a Cohort of Women Vulnerable to HIV in Alabama

**DOI:** 10.1093/ofid/ofag609

**Published:** 2026-09-26

**Authors:** Jiaying Hao, Desiree Phillips, Madeline C Pratt, Nuvan Rathnayaka, Kara Bennett, Anthony Merriweather, Lisa Hightow-Weidman, Henna Budhwani, Kathleen Hurwitz, Aadia I Rana, Latesha E Elopre, Lynn T Matthews

**Affiliations:** University of Alabama at Birmingham Heersink School of Medicine, Birmingham, Alabama, USA; University of Alabama at Birmingham Heersink School of Medicine, Birmingham, Alabama, USA; University of Alabama at Birmingham Heersink School of Medicine, Birmingham, Alabama, USA; Headwater Science, Durham, North Carolina, USA; Headwater Science, Durham, North Carolina, USA; Alabama Department of Public Health, Birmingham, Alabama, USA; College of Nursing, Florida State University, Tallahassee, Florida, USA; College of Nursing, Florida State University, Tallahassee, Florida, USA; Headwater Science, Durham, North Carolina, USA; University of Alabama at Birmingham Heersink School of Medicine, Birmingham, Alabama, USA; University of Alabama at Birmingham Heersink School of Medicine, Birmingham, Alabama, USA; University of Alabama at Birmingham Heersink School of Medicine, Birmingham, Alabama, USA; School of Medicine, Yale University, New Haven, Connecticut, USA

**Keywords:** HIV, STI, women, revuitment, data-based

## Abstract

The Camellia Cohort enrolls women with recent (past 90 days) gonorrhea or syphilis reported to the Alabama State Health Department. Eligible women are contacted to complete consenting procedures and home-based STI/HIV screening. Recruitment processes and strategies are refined based on interim findings, then modified to optimize recruitment yield.

Women in the Southern United States are disproportionately affected by HIV and other sexually transmitted infections (STIs). In 2022, of the 31 800 incident cases of HIV, women made up nearly 20%, with Southern women comprising almost half of cases [1]. HIV pre-exposure prophylaxis (PrEP) is highly effective in reducing HIV acquisition. Oral and long-acting injectable products are effective for women, but PrEP services are rarely tailored to the contextual and social background of Black women, and <2% of Black women in the United States use PrEP [2, 3]. Low uptake has been associated with medical mistrust, lack of knowledge about PrEP, and social and cultural stigma around PrEP and HIV [2-4].

To better understand factors that impact HIV incidence and prevention uptake among women in the Deep South, we are conducting the Camellia Cohort, a digital observational cohort study of women in Alabama with recent STI diagnoses. The Camellia Cohort utilizes a tailored smartphone app, HealthMpowerment (HMP) [5], and partners with the Alabama Department of Public Health (ADPH) to support women to complete home-based STI/HIV testing every 6 months and receive information about sexual health topics, including STIs and treatment, HIV, and PrEP. We share lessons learned from targeted recruitment activities for the first, to our knowledge, digital cohort of US Southern women to inform future work to optimize engagement and reach.

## METHODS

### Study Design and Population

Women aged 18–50, residing in Alabama, and diagnosed with gonorrhea or syphilis in the past 90 days are eligible to complete the screening procedures of consenting to and completing a home-based STI/HIV screening. Women who complete screening procedures and do not have HIV are eligible to enroll in the Camellia app and participate in study procedures (both app-based surveys and home-based STI/HIV testing) every 6 months.

### Recruitment and Research Methods

Collaborators at the ADPH STD Division obtained contact information for potentially eligible women through public health mandatory reporting systems. We used a stratified sampling framework with proportionate allocation to public health districts to contact eligible women via phone to introduce the Camellia Cohort study. Those who endorse interest complete consenting procedures for home-based STI/HIV screening and are mailed a kit through an existing home-based testing program at ADPH [6]. The kit includes a self-collected vaginal swab for *Neisseria gonorrhoeae*, *Chlamydia trachomatis*, and *Trichomonas vaginalis* (polymerase chain reaction testing), dried blood spot (DBS) test for *T. pallidum* antibodies (immunoglobulin G), and a combo antigen/antibody enzyme-linked immunosorbent assay for the qualitative detection of HIV p24 antigen and antibodies to HIV types 1 and 2 using the GS Combo Ag/Ab assay [7]. Participants who complete these steps and have a negative HIV test result are eligible to enroll. Participants complete a telephonic consenting process and are guided through the installation of the Camellia app on their mobile device.

Women could also self-refer through a QR code screening survey advertised on flyers and pocket cards distributed at community organizations and public health clinics statewide. Potentially eligible women were cross-referenced with ADPH surveillance data to confirm a recent gonorrhea or syphilis diagnosis and then contacted by study staff. Recruitment materials were developed with the Community Advisory Board to increase awareness and reach among potentially eligible women.

Recruitment metrics are tracked in REDCap [8, 9]; data abstraction from all participants in REDCap was conducted from November 2023 to January 2026. Recruitment outcomes are reported. A flowchart was generated to show the details of the recruitment steps and results (Figure 1).

**Figure 1.**
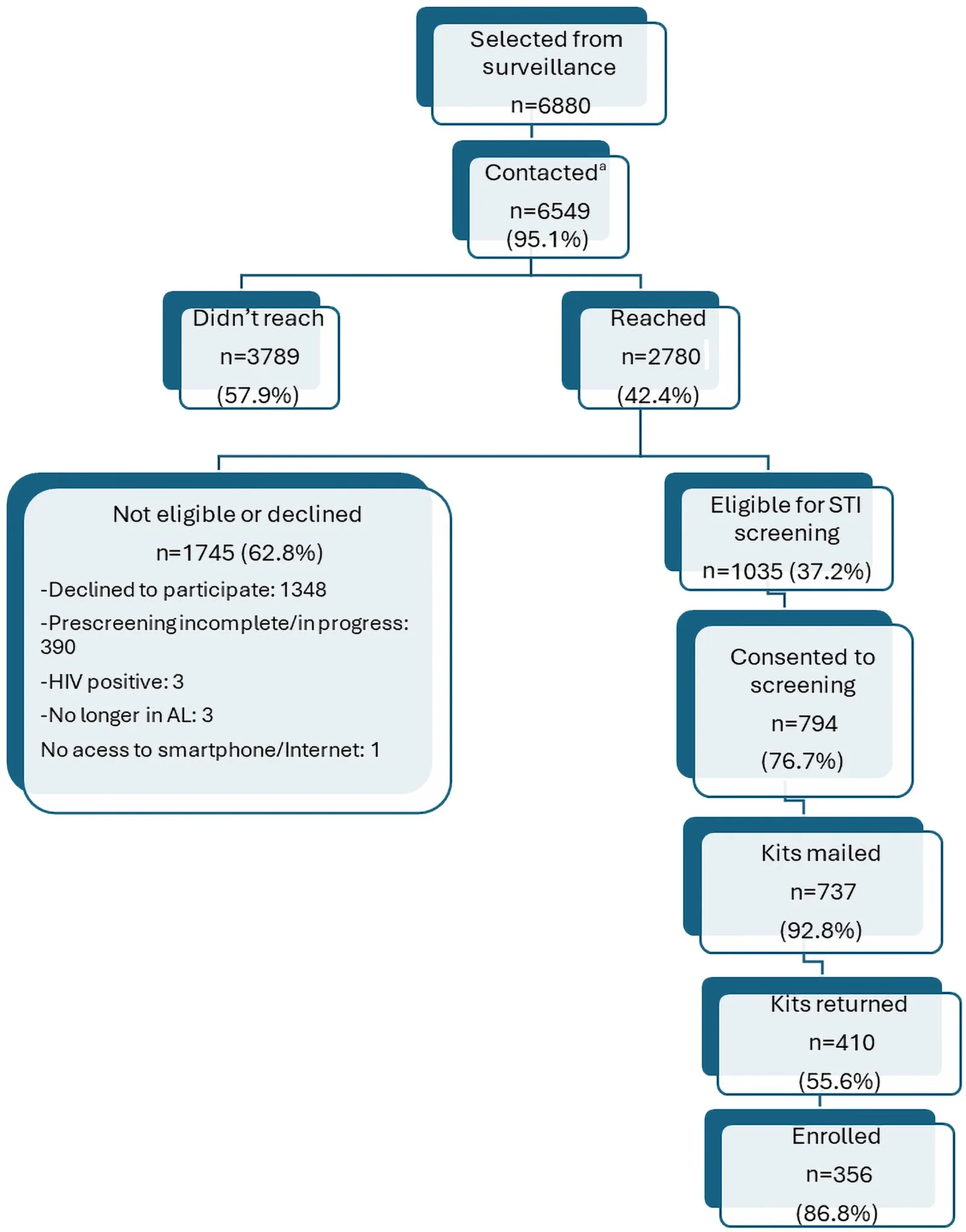
Recruitment flowchart. The flowchart shows the Camellia study recruitment and results from November 2023 to January 2026. The participants must be HIV-negative at initial STI screening. All positive STIs discovered through Camellia home-based testing were treated and followed up through ADPH standard of care. Special care was taken for clients with HIV-positive test results, with direct communication between study staff and ADPH leadership/disease intervention specialists to determine if diagnoses were newly diagnosed or known-positives. All HIV-positive tests through the program were determined to be previously known positives or false positives/unequivocal results that were confirmed negative with further testing. ^a^Contact was attempted in 6549 people; contact was not attempted in 311 people. Abbreviations: ADPH, Alabama Department of Public Health; STIs, sexually transmitted infections.

### Human Subjects/Ethical Review Information

The protocol was approved by the Institutional Review Board at University of Alabama at Birmingham (IRB-300009250) and the Research Review Committee at the ADPH (Montgomery, AL, USA). The protocol is registered at ClinicalTrials.gov (NCT 05463692).

## RESULTS

From November 2023 to January 2026, ADPH shared records for 10 921 women with recent syphilis or gonorrhea diagnoses. After age restrictions and removal of duplicates and enrolled participants, 6880 were potentially eligible. Of 6549 women contacted, 2780 (42.4%) were reached: 1786 (64.2%) on the first attempt, 721 (25.9%) on the second attempt, and 273 (9.8%) on the third attempt. Among 1035 women completing prescreening, 794 (76.7%) pre-enrolled for STI screening. Of 737 self-testing kits mailed, 410 (55.6%) were returned, and 356 women ultimately enrolled. Approximately 18 women needed to be contacted to enroll 1 participant. Table 1 shows demographic characteristics of potentially eligible and enrolled women.

**Table 1.** Demographic and Clinical Characteristics of Underlying, Eligible Population From ADPH and Enrolled Participants

	Eligible for Pre-enrollment STI Screening	Consented to STI Screening	No. Fully Eligible and Enrolled
	(n = 1035), No. (%)	(n = 794), No. (%)	(n = 356), No. (%)
Race/ethnicity	…	…	…
White/non-Hispanic	158 (17.6)	128 (18.7)	69 (21.9)
Black/other	738 (82.4)	557 (81.3)	246 (78.1)
Missing	139	109	41
Rurality	…	…	…
Metropolitan	869 (84.1)	672 (84.6)	295 (82.9)
Micropolitan	84 (8.1)	63 (7.9)	29 (8.1)
Small town	53 (5.1)	40 (5.0)	26 (7.3)
Isolated rural	27 (2.6)	19 (2.4)	6 (1.7)
Missing	2	0	0
Age	…	…	…
	25.0 [21.0–31.0]	26.0 [22.0–32.0]	27.0 [22.8–33.0]
Days since STI diagnosis	…	…	…
	46.0 [26.0–66.0]	45.0 [26.0–66.0]	46.0 [26.0–69.0]
STI at entry to ADPH database		…	…
Gonorrhea	853 (82.7)	647 (81.7)	289 (81.6)
Syphilis	0 (0)	0 (0)	0 (0)
Syphilis, early nonprimary, nonsecondary	57 (5.5)	44 (5.6)	14 (4.0)
Syphilis, primary	12 (1.2)	11 (1.4)	7 (2.0)
Syphilis, secondary	45 (4.4)	38 (4.8)	19 (5.4)
Syphilis, unknown duration or late	65 (6.3)	52 (6.6)	25 (7.1)

Median [25th percentile–75th percentile] is reported for age and days since STI diagnosis.

Abbreviations: ADPH, Alabama Department of Public Health; STI, sexually transmitted infection.

Among 484 women who self-referred through the online screener, 23 had a recent STI verified by ADPH, 9 met eligibility criteria, and 6 enrolled. Thus, ∼81 self-referrals were needed for 1 enrollment.

The largest drop in the recruitment cascade occurred at the contact stage, primarily due to an inability to reach potential participants via phone call (3789/6549, 57.9%). Common reasons for ineligibility among those successfully contacted included declining participation (1348/2780, 48.5%) in the prescreening phase and unreturned STI testing kits (327/737, 44.4%) among those who consented to screening. Reported barriers to kit return included difficulty conducting self-swabs and finger prick for sample collection, challenges completing the kits within the time window defined by the testing company (30 days), and challenges returning the kits via mail.

Based on participant feedback, staff experiences, and our Community Advisory Board, multiple adaptations were implemented to increase the number of participants recruited and optimize retention outcomes. To improve chances of successful contact on the first call, we added a texting/SMS option to alert participants to an incoming call that might be of interest. Third contact attempts during the initial outreach were low yield, leading the recruitment team to prioritize first and second contact attempts. To encourage women to return testing kits, we increased the associated incentive from $50 to $75 and offered telephone-based coaching. We also added mail pickup options for kit return to reduce logistical barriers encountered by rural participants.

## DISCUSSION

This study provides important operational details into remote recruitment and enrollment strategies for STI/HIV prevention research among women identified through public health surveillance systems. Despite access to a large pool of eligible participants, the recruitment cascade revealed substantial attrition, with the greatest loss occurring at the initial contact stage. More than half of eligible women could not be reached by phone, underscoring challenges in relying on telephone-based outreach. Inaccurate contact information and limited engagement with unknown callers [10] contribute to challenges contacting potential participants, which have been experienced by other digital cohorts [11].

The recruitment for home-based STI self-testing represented another key barrier, with nearly half of mailed kits not returned. Operational experience suggests that procedural complexity (eg, self-swabbing, finger-prick blood collection), time constraints, and logistical barriers such as limited access to shipping services may have contributed to noncompletion. Participants expressed the need for additional support from the study staff in relation to self-collection, including further instructional aids in the form of videos guiding the self-collection process and the ability for in-person dried blood spot testing. A similar study explored women-centered at-home testing for STIs. Participants discussed preferences for at-home STI testing and collecting their own vaginal swabs in comparison to having a health care provider collect samples [12]. These findings and ours align with prior literature indicating that while home-based testing can expand access, successful implementation requires robust participant support and simplified return processes [13–15].

Future analyses will evaluate changes in yield with these updates. The addition of SMS prenotification, reduction in call attempts, and expansion of community-based outreach reflect a shift toward more participant-centered and multimodal engagement strategies. The incorporation of self-referral pathways was implemented with an earlier analysis of our recruitment data. It is important to note that many of the self-referral clients believed they had had a recent curable STI, but there were no data in the public health system to verify recent syphilis or gonorrhea diagnoses. Many women do not know exactly what organism causes an STI infection—a recent diagnosis of trichomoniasis or herpes simplex or genital infections like candidiasis of bacterial vaginosis would not have been reported or made her eligible for this cohort [16]. This speaks to the challenges of relying on self-reports and the burden of documenting objective markers of disease for study enrollment. Although contributing to a small number of enrollments, the self-referral pathway remains a valuable complement to surveillance-based recruitment by providing an additional, participant-driven entry point. Enhancements to participant support—including increased incentive, telephone-based coaching, and flexible specimen return options—directly addressed identified barriers and are likely critical for improving enrollment and retention in similar studies [17].

## Limitations

Overall, these findings highlight that recruitment strategies leveraging public health data should be coupled with flexible, low-burden, and participant-informed approaches to maximize reach and enrollment. With self-collection kits, there is the possibility that study participants insufficiently collect their samples, causing a delay in the process of participants receiving their test results. Additionally, the stigma that is associated with HIV and receiving STI testing can deter potential participants from enrolling in the study. Future studies should continue to explore scalable strategies to improve contact rates and reduce barriers to home-based testing, particularly among populations disproportionately affected by STIs/HIV.

## References

[1] HIV.gov. US statistics. **2026**. Available at: https://www.hiv.gov/hiv-basics/overview/data-and-trends/statistics. Accessed February 5, 2026.

[2] Lockett A, Patterson E. The forgotten community: Black women weathering the waves of HIV. **2024**. Available at: https://oneill.law.georgetown.edu/the-forgotten-community-black-women-weathering-the-waves-of-hiv/. Accessed 1 September 2026.

[3] Randolph SD, Golin C, Welgus H, Lightfoot AF, Harding CJ, Riggins LF. How perceived structural racism and discrimination and medical mistrust in the health system influences participation in HIV health services for Black women living in the United States South: a qualitative, descriptive study. J Assoc Nurses AIDS Care 2020; 31:598–605. 10.1097/JNC.0000000000000189 32868634

[4] Campbell DM, Stockman JK. Black women in HIV research: intersectionality, positionality and our commitment to build a just research enterprise. Womens Health (Lond) 2024; 20:17455057241305071. 10.1177/17455057241305071 PMC11663266 39707883

[5] Muessig KE, Golinkoff JM, Hightow-Weidman LB, et al Increasing HIV testing and viral suppression via stigma reduction in a social networking mobile health intervention among Black and Latinx young men and transgender women who have sex with men (HealthMpowerment): protocol for a randomized controlled trial. JMIR Res Protoc 2020; 9:e24043. 10.2196/24043 PMC7773515 33325838

[6] ADPH OnImaware . 2026. Available at: https://adph.onimaware.com/. Accessed 1 September 2026.

[7] Pratt MC, Thomas TF, Merriweather A, et al Leveraging public health infrastructures to support sexual health for women living in the Southern United States. Front Med (Lausanne) 2025; 12:1521455. 10.3389/fmed.2025.1521455 PMC12436338 40963579

[8] Harris PA, Taylor R, Minor BL, et al The REDCap Consortium: building an international community of software platform partners. J Biomed Inform 2019; 95:103208. 10.1016/j.jbi.2019.103208 PMC7254481 31078660

[9] Harris PA, Taylor R, Thielke R, Payne J, Gonzalez N, Conde JG. Research Electronic Data Capture (REDCap)—a metadata-driven methodology and workflow process for providing translational research informatics support. J Biomed Inform 2009; 42:377–81. 10.1016/j.jbi.2008.08.010 PMC2700030 18929686

[10] Rufo Y . Why Gen Z & Millennials are hung up on answering the phone. **2024**. Available at: https://www.bbc.com/news/articles/crgklk3p70yo. Accessed 1 September 2026.

[11] Rendina HJ, Talan AJ, Tavella NF, et al Leveraging technology to blend large-scale epidemiologic surveillance with social and behavioral science methods: successes, challenges, and lessons learned implementing the UNITE longitudinal cohort study of HIV risk factors among sexual minority men in the United States. Am J Epidemiol 2021; 190:681–95. 10.1093/aje/kwaa226 PMC8024044 33057684

[12] Chow K, Edi R, Gin G, Morris SR. Attitudes of women participating in a clinical trial on point-of-care testing and home testing for STIs. Int J STD AIDS 2020; 31:1352–8. 10.1177/0956462420955067 32996866

[13] John SA, O'Neil AM, Baier V, et al HIV and syphilis self-testing to support the implementation of patient-delivered partner therapy for bacterial sexually transmitted infection partner treatment among sexual minoritized men. Sex Transm Dis 2026; 53:15–9. 10.1097/OLQ.0000000000002232 PMC12880594 40772618

[14] Kersh EN, Shukla M, Raphael BH, Habel M, Park I. At-home specimen self-collection and self-testing for sexually transmitted infection screening demand accelerated by the COVID-19 pandemic: a review of laboratory implementation issues. J Clin Microbiol 2021; 59:e0264620. 10.1128/JCM.02646-20 PMC8525576 34076475

[15] Sumray K, Lloyd KC, Estcourt CS, Burns F, Gibbs J. Access to, usage and clinic outcomes of, online postal sexually transmitted infection services: a scoping review. Sex Transm Infect 2022; 98:528–35. 10.1136/sextrans-2021-055376 PMC9613868 35701146

[16] Kowalczyk R, Nowosielski K. Impact of social factors and sexual behaviors on the knowledge of sexually transmitted infections among women who have sex with women/women who have sex with women and men. Int J STD AIDS 2019; 30:163–72. 10.1177/0956462418802736 30348067

[17] Gurung S, Jones SS, Mehta K, et al Examining recruitment strategies in the enrollment cascade of youth living with HIV: descriptive findings from a nationwide web-based adherence protocol. JMIR Form Res 2023; 7:e40077. 10.2196/40077 PMC10131637 36745773

